# Prognostic Value of Blood Count after Haemorrhage and Transfusion

**Published:** 1918-05

**Authors:** 


**Prognostic Value of Blood Count After Haemorrhage and Transfusion.** Depage and Govaerts, (Belgian Army), Le Prog. Med., Nov. 10, 1917, state their experience to be that, after a haemorrhage sufficiently large to endanger life, and transfusion, if the red cells do not rise above 4 million in the venous blood within 6 hours, a fatal prognosis is almost always justified. Injections of artificial serum are not considered of value.

